# Die NVL Depression benötigt mutigere und differenziertere Aussagen zur Psychotherapie

**DOI:** 10.1007/s00115-022-01351-w

**Published:** 2022-07-14

**Authors:** Winfried Rief, Eva-Lotta Brakemeier, Tim Kaiser, Tilo Kircher, Klaus Lieb, Jürgen Margraf, Johannes Michalak, Andreas Reif, Silvia Schneider, Ulrich Voderholzer

**Affiliations:** 1 Klinische Psychologie und Psychotherapie, Universität Marburg, Gutenbergstr. 18, 35032 Marburg, Deutschland; 2 Klinische Psychologie und Psychotherapie, Zentrum für Psychologische Psychotherapie (ZPP), Universität Greifswald, Greifswald, Deutschland; 3 Universität Greifswald, Greifswald, Deutschland; 4 Klinik für Psychiatrie und Psychotherapie, Universitätsmedizin Mainz, Mainz, Deutschland; 5 Forschungs- und Behandlungszentrum für psychische Gesundheit (FBZ), Ruhr-Universität Bochum, Bochum, Deutschland; 6 Department für Psychologie und Psychotherapie, Lehrstuhl für Klinische Psychologie und Psychotherapie II, Universität Witten-Herdecke, Witten, Deutschland; 7 Klinik für Psychiatrie, Psychosomatik und Psychotherapie, Universitätsklinikum Frankfurt, Frankfurt am Main, Deutschland; 8 Schoen Klinik Roseneck, Prien a. Ch., Deutschland

Eine Leitlinie soll Fachpersonen, Betroffenen und der gesamten Bevölkerung evidenzbasierte Empfehlungen auf Basis des aktuellen wissenschaftlichen Erkenntnisstands zu Diagnostik- und Behandlungsmöglichkeiten geben. Der 3. Entwurf zur Nationalen Versorgungsleitlinie (NVL) Depression^1^ wird aktuell zur Diskussion gestellt. Gerade die Diskussionsvorlage zeigte, dass die neue NVL diesem Auftrag der systematischen und differenzierten Informationsvermittlung an verschiedenen Stellen nicht ausreichend nachkommt, was im Bereich Psychotherapie besonders deutlich wird. Die wichtigsten Kritikpunkte sind:

## Wissenschaftliche Bewertung der Effektivität verschiedener Methoden

Bereits jetzt werden in Deutschland unterschiedliche psychotherapeutische Behandlungsansätze in der ambulanten und stationären Versorgung umgesetzt, sodass sich die Frage nach der aktuellen wissenschaftlichen Bewertung der Methoden stellt – eine Antwort auf diese Frage findet sich in der NVL Depression leider nicht. Die enorme Forschungsdynamik mit aktuell über 200 Psychotherapie-RCTs (Randomisierte klinische Studien) pro Jahr [17] bleibt unberücksichtigt. Zu Beginn des Berichtes (S. 17) wird festgelegt, dass nach Ansicht der Autorinnen und Autoren die Hauptwirkung von Psychotherapie auf allgemeine Faktoren („common factors“) zurückzuführen ist und Äquivalenz der Ansätze angenommen wird. Dabei wird nicht berücksichtigt, dass vermeintlich vergleichbare Wirksamkeit zum Teil auf statistischen Artefakten basieren kann [13]. Die NVL bestätigt zwar, dass die Wirksamkeitsbelege für die einzelnen Psychotherapieverfahren sehr unterschiedlich sind und dass nicht jedes Verfahren und jede Methode für jede Patientin und jeden Patienten geeignet ist (S. 85), jedoch wird über diese Unterschiede in der wissenschaftlichen Untermauerung von Behandlungsansätzen bei einzelnen Untergruppen der Depression nicht informiert. Dosis-Wirkungs-Zusammenhänge, Unterschiede in der Therapiedichte und gesundheitsökonomische Bewertungen werden in die Empfehlungen nicht einbezogen. Der Zeitpunkt zum Erreichen des primären Endpunkts ist jedoch ein sehr relevanter und zusätzlich zur Effektstärke darzustellender Faktor, da es für Betroffene von großer Bedeutung ist, ob die Remission nach drei oder zwölf Monaten erfolgt. Eine aussagekräftige Aufwand-Risiko-Nutzen-Bewertung unterbleibt.

## Wirkfaktoren der Psychotherapie

Bei der Diskussion von Wirkfaktoren werden in der NVL lang bekannte Annahmen in den Vordergrund gestellt („Beziehung ist wichtig“), ohne darauf hinzuweisen, dass diese Aussage bei der Psychotherapie primär auf korrelativen Studien beruht, die erklärte Varianz der Ergebnisse oft unter 10 % liegt [8, 12] und die Kausalität in vielen Fällen auch umgekehrt sein kann: Eine gute Allianz kann auch *Folge* einer erfolgreichen, am akuten Problem orientierten Therapie sein [9]. Außerdem handelt es sich hier um einen Faktor, wie man ihn ähnlich auch bei medikamentösen Interventionen findet, sodass der eigentliche Erklärungswert gering ist [16, 19]. Demgegenüber werden empirisch gut gesicherte und spezifische Wirkfaktoren (z. B. Aufbau von Aktivität [7, 15]) kaum erwähnt.

## Generalisierte Gleichsetzung aller Richtlinienverfahren

Die NVL orientiert sich stark an den Richtlinienverfahren (also systemische Therapie, tiefenpsychologische Therapie, analytische Psychotherapie und kognitive Verhaltenstherapie); verfahrensübergreifende innovative Ansätze werden kaum vertieft bzw. nur oberflächlich den Richtlinienverfahren zugeordnet. Am Beispiel leichter Depressionen wird andiskutiert, dass zwischen den Richtlinienverfahren bisher kein signifikanter Unterschied in der Wirksamkeit belegt wurde. Aus dieser Diskussion schlussfolgert die NVL, dass auch bei allen anderen Einzelindikationen vermutlich kein signifikanter Unterschied gefunden würde (z. B. bei schweren Depressionen, chronischen Depressionen oder Rückfallverhinderung), selbst dann, wenn es für das einzelne Richtlinienverfahren bei den genannten spezifischen Indikationen keine belastbare Evidenz gibt. Dies ist eine bei Leitlinienentwicklungen kaum akzeptable Generalisierung und verhindert eine sachliche Information der Bevölkerung.

Liegen für Therapieverfahren wenig Studien vor, sind die Konfidenzintervalle der zu erwartenden Effekte sehr groß und es bleibt oftmals unklar, ob diese Interventionen einerseits wirklich über einfache Placeboeffekte hinausgehen oder andererseits vielleicht sogar bessere Effekte als bei Vergleichstherapien zu erwarten sind [2]. Unterschiede in Remissionsraten oder fehlende Evidenz für positive Langzeiteffekte [4] einzelner Therapieansätze bei bestimmten Indikationsbereichen bleiben in der NVL unkommentiert.

## Evidenz psychotherapeutischer Ansätze bei Untergruppen der Depressionen

Auch die an anderer Stelle immer wieder vorgenommene Aufteilung in leichtgradige, mittelschwere und schwere Depressionen wird für die Psychotherapieforschung nicht durchkonjugiert. Es gibt durchaus Hinweise, dass Therapieverfahren in Abhängigkeit von der Schwere und dem Verlauf der Störung unterschiedlich wirksam sind (z. B. [20]). Wenn die NVL bei mittelschweren Depressionen die Empfehlung zur Psychotherapie gibt und vorgibt, dass diese gleichwertig zur Pharmakotherapie ist (Empfehlungen 5 bis 8), gilt dies dann wirklich für alle Richtlinientherapien gleichermaßen? Wenn bei schweren Depressionen in der NVL die Behandlungsempfehlung gegeben wird, dass eine Kombinationstherapie mit Antidepressiva und Psychotherapie besser wäre als Monotherapie, gilt dies dann auch für eine Kombination aus Langzeitpsychoanalyse mit Antidepressiva? Wie sieht es mit episodisch wiederkehrenden vs. chronisch-persistierenden Depressionen aus, haben alle Psychotherapieverfahren für beide Varianten vergleichbare Wirknachweise [14]? Ist für alle Psychotherapieansätze wirklich gleich robust belegt, dass sie präventiv und rückfallverhindernd wirken, wie dies einige Ansätze nachweisen konnten [14]? Über die wissenschaftlichen Antworten auf diese Fragen wird die Bevölkerung durch die NVL nicht informiert.

## Das Beispiel NICE-Guidelines

Im Gegensatz zu der NVL haben die britischen wissenschaftlich fundierten Empfehlungen der NICE-Guidelines offensichtlich den Mut aufgebracht, darauf hinzuweisen, dass nicht alle Psychotherapieansätze gleiche Evidenz haben. Die Bevölkerung wird informiert, welche Psychotherapieansätze bei leichten, bei mittelschweren und bei schweren Depressionen Wirkungsbelege haben, welche zur Prävention von Rückfällen bei rekurrenten Formen von Depressionen Wirkungsnachweise haben etc. Dieser Informationspflicht sollte auch die NVL Depression nachkommen.

## Einsatz psychometrischer Verfahren

Für den Einsatz psychometrischer Verfahren werden nur zögerliche Empfehlungen für die Routineversorgung gegeben. „Aus Sicht der Leitliniengruppe sind die meisten psychometrischen Depressionsskalen außerhalb spezialisierter Einrichtungen oder wissenschaftlicher Analysen jedoch aufgrund ihrer Komplexität nur bedingt geeignet“ (S. 43). Dies missachtet, dass Experteneinschätzungen gerade im Depressionsbereich starken Erwartungseffekten unterliegen [3, 18] und deshalb ein psychometrisches Instrument eine wertvolle Zusatzinformation insbesondere zu Schwere und Verlauf erbringt. Dass die wenigen Minuten Aufwand hierfür „zu komplex“ wären, ist in Anbetracht der sonst in der Medizin üblichen Aufwände zur Verbesserung der Diagnostik und Verlaufskontrolle wenig stichhaltig. „Eine unwirksame Behandlung beizubehalten, ist ethisch unvertretbar“ (S. 86). Diese unterstützenswerte Aussage erfordert Veränderungsbewertungen, die nicht nur auf der subjektiven Meinung des Behandelnden beruhen. Diesbezüglich ist Empfehlung 4.29 zu schwach. Es gibt sehr viel Evidenz, dass sich durch das Hinzuziehen psychometrischer Instrumente die Validität der Bewertung substanziell und signifikant verbessert [1, 10, 11] und durch psychometrisch gestützte Rückmeldesysteme Behandlungsverläufe effektiver werden (s. z. B. IAPT [6]). Dadurch können Abbruchraten gesenkt und Therapieerfolge vergrößert werden [5]. Kaum jemand würde in der Diagnostik chronischer Schmerzen oder in der Demenzdiagnostik postulieren, dass der klinisch erfasste Eindruck des Behandelnden allein ausreichend ist zur Verlaufsbeurteilung der Symptomatik und Funktionalität. Die NVL ist hier bedrohlich nahe an Empfehlungen, die Fehldiagnostik und ungünstige Behandlungsverläufe fördern.

## Empfehlung

Viele der genannten Kritikpunkte haben bereits vorherige Versionen der NVL Depression gekennzeichnet, manche sind neu. Die Leitlinie enthält viele subjektive Interpretationen und a priori Setzungen, die nicht wissenschaftlich abgesichert sind. Andere, wissenschaftlich abgesicherte Befunde werden in der Bedeutung nivelliert. An diversen Stellen priorisiert die NVL die aktuelle Versorgung durch die Richtlinienverfahren, anstatt neue, wissenschaftlich fundierte Impulse zur Veränderung zu geben. Analog zu pharmakologischen Empfehlungen sollte das deutsche System der Richtlinienpsychotherapie keine Bedeutung haben für die wissenschaftliche Bewertung von Therapiemethoden; ganz im Gegenteil sollte es Aufgabe der Leitlinie sein, das Versorgungssystem auf der Basis der Evidenz zu verändern. Vor diesem Hintergrund empfiehlt sich ein kompletter Neuentwurf der Leitlinie, der konsequent an der wissenschaftlichen Evidenz und wissenschaftlichen Dynamik orientiert ist und über die Befundlage differenziert informiert, auch und gerade über Unterschiede in der Evidenz. Wenn dieser mutige Schritt einer Leitlinie aus Gründen der Konfliktvermeidung nicht gemacht wird, erscheint eine Leitlinie wenig hilfreich.

## Footnotes

1 Trotz der hier vorgetragenen Kritik, die sich am Diskussionsvorschlag zur NVL festmacht, gebührt unser Dank allen Personen, die an der Überarbeitung zur 3. Version der NVL Depression mitgewirkt haben. Unter hohem Zeitaufwand geschieht dies in aller Regel ehrenamtlich ohne besondere Entlohnung, sodass dieses Engagement ausgesprochen anerkennenswert ist.
